# The cost-effectiveness of tracking newborns with bilateral hearing impairment in Bavaria: a decision-analytic model

**DOI:** 10.1186/1472-6963-12-418

**Published:** 2012-11-22

**Authors:** Astrid Langer, Inken Brockow, Uta Nennstiel-Ratzel, Petra Menn

**Affiliations:** 1 Section Quality of Health Care, Health Economics, Health System Analysis, Bavarian Health and Food Safety Authority, Eggenreuther Weg 43, 91058, Erlangen, Germany; 2 Institute of Health Economics and Health Care Management, Munich School of Management, Ludwig-Maximilians-Universität München, Ludwigstraße 28, 80539, Munich, Germany; 3 Institute of Health Economics and Health Care Management, Helmholtz Zentrum München – German Research Centre for Environmental Health, Ingolstädter Landstraße 1, 85764, Neuherberg, Germany; 4 Screening Centre of the Bavarian Health and Food Safety Authority, Veterinärstr. 2, 85764, Oberschleißheim, Germany

**Keywords:** ‘Neonatal Screening’[Mesh], ‘Hearing Disorders’[Mesh], ‘Costs and Cost Analysis’[Mesh], ‘Decision Support Techniques’[Mesh], ‘Germany’[Mesh]

## Abstract

**Background:**

Although several countries, including Germany, have established newborn hearing screening programmes for early detection and treatment of newborns with hearing impairments, nationwide tracking systems for follow-up of newborns with positive test results until diagnosis of hearing impairment have often not been implemented. However, a recent study on universal newborn hearing screening in Bavaria showed that, in a high proportion of newborns, early diagnosis was only possible with the use of a tracking system. The aim of this study was, therefore, to assess the cost-effectiveness of tracking newborns with bilateral hearing impairment in Bavaria.

**Methods:**

Data from a Bavarian pilot project on newborn hearing screening and Bavarian newborn hearing screening facilities were used to assess the cost-effectiveness of the inclusion of a tracking system within a newborn hearing screening programme. A model-based cost-effectiveness analysis was conducted. The time horizon of the model was limited to the newborn hearing screening programme. Costs of the initial hearing screening test and subsequent tests were included, as well as costs of diagnosis and costs of tracking. The outcome measure of the economic analysis was the cost per case of bilateral hearing impairment detected. In order to reflect uncertainty, deterministic and probabilistic sensitivity analyses were performed.

**Results:**

The incremental cost-effectiveness ratio of tracking vs. no tracking was €1,697 per additional case of bilateral hearing impairment detected.

**Conclusions:**

Compared with no tracking, tracking resulted in more cases of bilateral hearing impairment detected as well as higher costs. If society is willing to pay at least €1,697 per additional case of bilateral hearing impairment detected, tracking can be recommended.

## Background

In Germany, the prevalence of congenital hearing impairments is approximately 1.2 cases per 1,000 newborns [1]. Newborn hearing screening is used ‘to identify hearing impairments shortly after birth to initiate treatment as soon as possible and to allow affected children to enjoy largely normal development’ (p. 130) [2]. A systematic review of newborn hearing screening by Wolff et al. [2] found that screening, and earlier treatment, were both associated with better language development. These findings are supported by a recent review [3].

In Germany, newborn hearing screening for the early detection of hearing impairment was legally mandated in 2008 and came into effect on 1 January 2009 [4]. Since then, all newborns insured by Statutory Health Insurance in Germany have been entitled to newborn hearing screening. In Germany, the primary aim of newborn hearing screening is to detect bilateral hearing impairments of 35 dB or more (using transient evoked otoacoustic emissions (TEOAE) or automated auditory brainstem response (AABR) in the first stage and AABR in the second stage) in the first three months of life, and to initiate treatment in the first six months of life. However, a nationwide tracking system – that is, a nationwide system to ensure completeness of participation and the follow-up of newborns with positive (conspicuous) hearing (screening) test results until diagnosis of hearing impairment – was not included [4].

Several research groups have recently endorsed the need for a tracking system. For instance, Rohlfs et al. argue that ‘the implementation of newborn hearing screening only makes sense if there exists an efficient tracking system’ (p. 1453) [5]. In its report ‘Newborn hearing screening in the detection of hearing impairment’, the Institute for Quality and Efficiency in Health Care found that, for early diagnosis and treatment, the full-scale implementation of newborn hearing screening may not be sufficient. They argue that substantial benefit from newborn hearing screening can only be expected if screening is reinforced by organizational structures which ensure there are neither delays nor disruptions from the point of initial suspicion of hearing impairment to its subsequent treatment [1]. In a recent review, Ptok points out that ‘the most important single measure for the practical realization of early detection of hearing impairments in newborns and infants in Germany seems to be the installation of a system of tracking centers covering the whole country’ (p. 430) [6]. Furthermore, a recent study on universal newborn hearing screening two years after its full-scale implementation in Bavaria showed that, in 49% of newborns with hearing impairment, early diagnosis was only possible through the use of a tracking system [7]. Therefore, in the absence of a tracking system for follow-up of newborns with positive (conspicuous) hearing (screening) test results until diagnosis of hearing impairment, early suspicion of hearing impairment may not actually result in early detection and treatment.

The aim of this study, therefore, was to assess the cost-effectiveness of tracking newborns with bilateral hearing impairment in Bavaria based on data from Bavarian newborn hearing screening facilities in a decision-analytic model.

## Methods

### Decision-analytic model: scope and perspective

In Germany, a nationwide newborn hearing screening programme was implemented in 2009, but a tracking system covering the whole country was not considered. In May 2003, a newborn hearing screening programme including a tracking system was initiated in the Upper Palatinate based on an interdisciplinary design [8]. In the pilot project, referred to as ‘newborn auditory screening’, consecutive TEOAE and AABR screening tests were conducted. The Screening Centre at the Public Health Authority was involved in coordinating the screening process. It was responsible for the completeness of participation, the follow-up of newborns with a positive screening test result, and quality assurance of the pilot project. For this purpose, the screening centre maintained registers of newborns who had not been screened and newborns with a positive (conspicuous) screening test result. The tracking system established at the screening centre was used, on the one hand, to achieve a high coverage rate, i.e. a high proportion of newborns screened, and on the other hand a low loss to follow-up rate for subsequent tests. The interventions of the tracking system included recalls by letter and telephone and, if required, involvement of the public health office. Data from the pilot project and Bavarian newborn hearing screening facilities were, therefore, used to assess the cost-effectiveness of the inclusion of a tracking system within a newborn hearing screening programme. These data were obtained from the Bavarian Food and Health Safety Authority and some of them are publicly available. A model-based cost-effectiveness analysis was conducted from the perspective of the German Statutory Health Insurance, taking into account the costs of tracking. Owing to a lack of long-term data, the time horizon of the model was limited to the newborn hearing screening programme (initial hearing screening test and subsequent hearing tests or diagnosis). Therefore, discounting was not relevant. The model included the costs of the initial hearing screening test and subsequent hearing tests, costs of diagnosis, and costs of tracking. Only bilateral hearing impairment of 35 dB or more was considered, as is standard practice in Germany. Moreover, there is a lack of evidence concerning the benefits of early detection in newborns with unilateral hearing impairment in terms, for example, of language and speech development [9]. Therefore, the outcome measure of the economic analysis was the cost per case of bilateral hearing impairment detected.

There are several good-practice guidelines for decision-analytic modelling in health economic evaluation [10-16]. The decision-analytic model developed here follows the guidelines established by Philips et al. [10], as these are the result of a review and synthesis of existing good practice guidelines. With regard to the model’s technical documentation, the guidelines on modelling provided by the Institute on Quality and Efficiency in Health Care were followed [16]. TreeAge Pro 2011 software was used to build a static decision tree model, which is appropriate for the analysis of the probabilities of events characterized by limited change or recurrence over time, such as the probability that a newborn is hearing impaired or not [17-19].

### Decision-analytic model: structure

**Figure 1:**
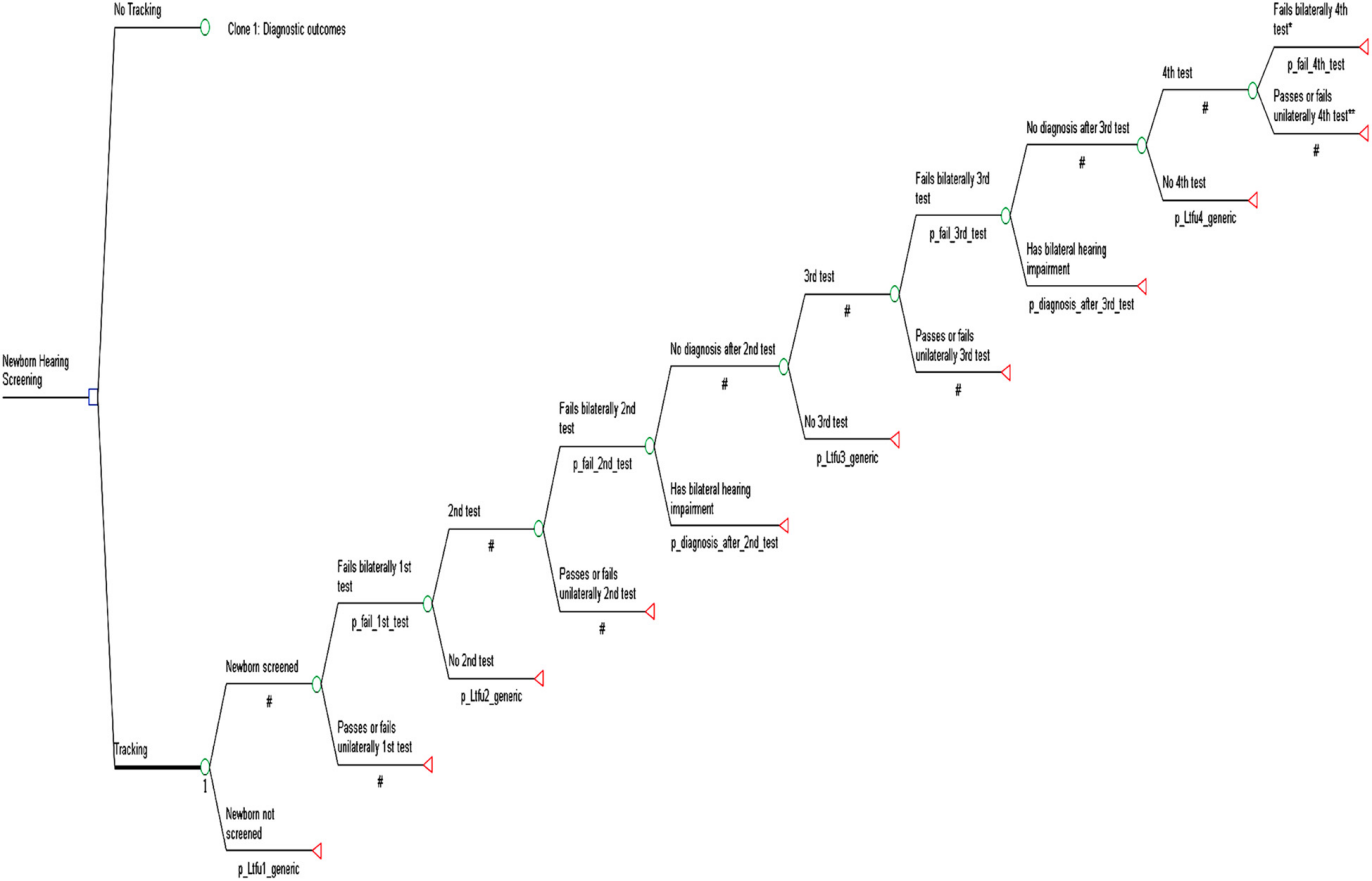
Structure of the model.

The structure of the decision tree is largely based on the test procedure of the pilot project ‘newborn auditory screening’ [8]. The structure of the decision tree was also informed by existing decision tree models concerning newborn hearing screening [20,21] and is shown in Figure 1. In Germany, a two-stage newborn hearing screening programme performed in hospital before discharge should immediately be followed by confirmatory diagnostic evaluation [4]. As there are not enough pediatric audiologists or otolaryngologists with expertise in phoniatry and pedaudiology to perform confirmatory diagnostic evaluation, in the pilot project ‘newborn auditory screening’, up to two other hearing tests (AABR, otoacoustic emissions (OAE), or both OAE and AABR) were performed after discharge from hospital, and before referral to pediatric audiologists or otolaryngologists with expertise in phoniatry and pedaudiology for confirmatory diagnostic evaluation. Therefore, the decision tree includes a four-stage test procedure for newborns that bilaterally fail the first, second, and third hearing tests – that is to say, newborns who have a positive bilateral hearing impairment test result are scheduled for an additional hearing test. Whereas the first hearing test is a two-stage screening procedure (first TEOAE or AABR and then, if the TEOAE or AABR screening test result is positive, AABR), the other hearing tests are one-stage tests using OAE, AABR, or both OAE and AABR. Newborns do not run through the four-stage test procedure in the following four cases: they are not screened; they are lost to follow-up after the first, second, or third test; they pass the first, second, or third test (i.e. have no evidence of bilateral hearing impairment); or they unilaterally fail the first, second, or third test. Newborns with bilateral hearing impairment in this group may be identified at a later date outside the newborn hearing screening programme, for example due to parental concern. As these newborns are not identified at an early stage as a consequence of screening, they are not counted as part of the yield of the four-stage test procedure [20].

### Decision-analytic model: variable estimation

Table 1 gives the probabilities of events related to the four-stage test procedure. These probabilities were taken from published and unpublished data in the pilot project ‘newborn auditory screening’ [8] and, where data from the pilot project were not available, from Bavarian newborn hearing screening facilities for 2010. The data include the probability that the newborn is lost to follow-up before the first test (i.e. the probability that the newborn is not screened) or to subsequent tests, with and without tracking; that it bilaterally fails the first or subsequent tests; and that it is diagnosed after the second or third test. The probability of failing the first test was estimated from the number of newborns who fail the first test divided by the number of newborns screened. The probability of failing subsequent tests was conditional on having failed previous tests.

**Table 1:** Probabilities of events related to the four-stage test procedure

**Name**	**Description**	**Mean (95% CI)**	**Source**
p_Ltfu1_t	Probability of loss to follow-up before the initial hearing screening test with tracking	0.045 (0.043; 0.047)	Pilot project ‘newborn auditory screening’ [8]
p_Ltfu1_nt	Probability of loss to follow-up before the initial hearing screening test without tracking	0.053 (0.051; 0.055)	Pilot project ‘newborn auditory screening’ [8]
p_Ltfu2_t	Probability of loss to follow-up before 2nd test with tracking	0.07 (0.057; 0.084)	Data from the state-wide screening programme for 2010
p_Ltfu2_nt	Probability of loss to follow-up before 2nd test without tracking	0.51 (0.497; 0.524)	Data from the state-wide screening programme for 2010
p_Ltfu3_t	Probability of loss to follow-up before 3rd test with tracking	0.08 (0.048; 0.117)	Data from the state-wide screening programme for 2010
p_Ltfu3_nt	Probability of loss to follow-up before 3rd test without tracking	0.29 (0.256; 0.325)	Data from the state-wide screening programme for 2010
p_Ltfu4_t	Probability of loss to follow-up before 4th test with tracking	0.07 (0.027; 0.133)	Data from the state-wide screening programme for 2010
p_Ltfu4_nt	Probability of loss to follow-up before 4th test without tracking	0.18 (0.133; 0.239)	Data from the state-wide screening programme for 2010
p_fail_1st_test	Probability of failing the initial hearing screening test bilaterally	0.006 (0.005; 0.007)	Pilot project ‘newborn auditory screening’ [8]
p_fail_2nd_test	Probability of failing the 2nd test bilaterally	0.202 (0.167; 0.241)	Pilot project ‘newborn auditory screening’*
p_fail_3rd_test	Probability of failing the 3rd test bilaterally	0.627 (0.512; 0.739)	Pilot project ‘newborn auditory screening’*
p_fail_4th_test	Probability of failing the 4th test bilaterally	0.609 (0.400; 0.785)	Pilot project ‘newborn auditory screening’*
p_diagnosis_after_2nd_test	Probability of diagnosis after 2nd test	0.11 (0.073; 0.159)	Data from the state-wide screening programme for 2010
p_diagnosis_after_3rd_test	Probability of diagnosis after 3rd test	0.36 (0.261; 0.460)	Data from the state-wide screening programme for 2010

*unpublished data.

Table 2 shows the cost items used to calculate the costs of hearing (screening) tests and diagnosis, and Table 3 provides the costs of the initial hearing screening test, subsequent tests, and diagnosis. It is assumed that the first test – that is, the initial hearing screening test – is performed in a hospital, while the second and third tests are performed by office-based ear, nose, and throat (ENT) physicians. Newborns who fail the second, third, or fourth test by pediatric audiologists receive a final diagnosis. Office-based ENT physicians and office-based pediatric audiologists receive a specific lump sum in addition to the costs of the tests. In order to confirm or exclude bilateral hearing impairment, pediatric audiologists perform AABR and an impedance test of the middle ear. As inpatient services in Germany are generally reimbursed as a lump sum by diagnosis-related groups (DRGs), costs for inpatient tests (i.e. the first test) were estimated based on the tariff scheme of the German Hospital Federation (DKG-NT [22]). Costs for outpatient tests (i.e. the second, third, and fourth tests), were taken from the doctor’s fee scale (EBM [23]). All costs were calculated as the product of points and point value (point price). The costs for tests were weighted by the probability that a newborn receives OAE, AABR, or both OAE and AABR. These probabilities were taken from the pilot project and based on assumptions. Unlike the other tests, the first test is a two-stage screening test: in the first stage, 95.2% of newborns are screened by OAE and 4.8% by AABR; in the second stage, all newborns who bilaterally fail the initial screening test (7.66%) are screened by AABR. The probability of using both OAE and AABR was 10.4% for the second test, 20.9% for the third test, and 0% for diagnostic tests performed by pediatric audiologists. The probability of using OAE or AABR was respectively 74.7% or 14.9% for the second test, 40.3% or 38.8% for the third, and 0% or 100% for tests performed by pediatric audiologists. The costs of tests were €42.22 for the first test, €38.99 for the second test, €43.39 for the third test, and €91.83 for diagnostic tests performed by pediatric audiologists. The costs of tracking were taken from the pilot project ‘newborn auditory screening’ and were calculated at €4.55 per newborn screened (total costs of tracking divided by the number of newborns screened). A detailed breakdown of the costs of tracking is provided in Table 4.

**Table 2:** Cost items used to calculate the costs of hearing (screening) tests and diagnosis

**Name**	**Description**	**Item**	**Points**	**Point value**	**Mean**	**Source**
c_OAE_inpatient	Costs of OAE performed in hospital	1409	400	8.589704 Cent	€34.36	DKG-NT [22]
c_AABR_inpatient	Costs of AABR performed in hospital	1408	888	8.589704 Cent	€76.28	DKG-NT [22]
c_lumpsum_ENT	Lump sum for office-based ENT physicians	09210	680	3.5048 Cent	€23.83	EBM [23]
c_OAE_outpatient_ENT	Costs of OAE performed by office-based ENT physicians	09324	340	3.5048 Cent	€11.92	EBM [23]
c_AABR_outpatient_ENT	Costs of AABR performed by office-based ENT physicians	01706	705	3.5048 Cent	€24.71	EBM [23]
c_lumpsum_PA	Lump sum for office-based pediatric audiologists	20210	865	3.5048 Cent	€30.32	EBM [23]
c_AABR_outpatient_PA	Costs of AABR performed by office-based pediatric audiologists	20327	1535	3.5048 Cent	€53.80	EBM [23]
c_MEM_reflex_outpatient_PA	Costs of impedance test of the middle ear performed by office-based pediatric audiologists	20323	220	3.5048 Cent	€7.71	EBM [23]

AABR: automated auditory brainstem response; CI: confidence interval; DKG-NT: ‘Deutsche Krankenhausgesellschaft-Normaltarif’; EBM: ‘Einheitlicher Bewertungsmaßstab’; ENT: ear, nose, and throat; MEM: middle ear muscle; OAE: otoacoustic emissions; PA: pediatric audiologist.

**Table 3:** Costs of the hearing screening test, subsequent tests and diagnosis

**Name**	**OAE (in %)**	**AABR (in %)**	**Both, OAE and AABR (in %)**	**Mean**	**Comment**
c_1st_test_2stages^a^	95.2^e^/0^f^	4.8^e^/7.66^f^	0^e^/0^f^	€42.22	(0.952*c_OAE_inpatient+0.048*c_AABR_inpatient)+0.0766*c_AABR_inpatient
c_2nd_test_1stage_ENT^b^	74.7	14.9	10.4	€38.99	(0.104^g^+0.149)*c_AABR_outpatient_ENT+0.747*c_OAE_outpatient_ENT+c_lumpsum_ENT
c_3rd_test_1stage_ENT^c^	40.3	38.8	20.9	€43.39	(0.209^g^+0.388)*c_AABR_outpatient_ENT+0,403*c_OAE_outpatient_ENT+c_lumpsum_ENT
c_test_PA^d^	0	100	0	€91.83	c_lumpsum_PA+c_MEM_reflex_outpatient_PA+c_AABR_outpatient_PA

^a^Costs of the initial two-stage hearing screening test performed in hospital; ^b^Costs of 2nd test performed by office-based ENT physicians; ^c^Costs of 3rd test performed by office-based ENT physicians; ^d^Costs of confirmatory diagnostic testing performed by pediatric audiologists; ^e^1st stage; ^f^2nd stage; ^g^Costs of OAE are included in costs of AABR; AABR: automated auditory brainstem response, ENT: ear, nose, and throat, MEM: middle ear muscle, OAE: otoacoustic emissions, PA: pediatric audiologist.

**Table 4:** Costs of tracking from the pilot project ‘newborn auditory screening

**Personnel expenses**	
Nurse (0.75 position; tariff part: E8)	€33,876
Epidemiologist (0.5 position; tariff part: E14)	€31,759
*Personnel expenses total*	*€65,635*
Non-personnel expenses	
Printing costs (flyer, data sheets)	€1,500
Stamped addressed envelopes for hospitals and doctors’ offices (50 per week)	€1,590
Telephone/fax (40 per working day)	€1,000
Room	€4,200
Two desktop PCs	€3,000
One telephone (nationwide connection)	€100
Letters (eight per working day)	€1,000
*Non-personnel expenses total*	*€12,390*
**Total costs**	**€78,025**

### Decision-analytic model: uncertainty and consistency

The decision-analytic model was developed and validated by discussion with experts in the provision of newborn hearing screening. In order to reflect uncertainty, both deterministic and probabilistic sensitivity analyses were performed to show how the model’s outputs change with variation in its inputs. In univariate sensitivity analyses, parameters were varied by ±50%. A beta distribution was assumed for all probabilities and a gamma distribution for all cost parameters [17]. A structural sensitivity analysis was also performed to analyse how the results change with a variation in the test procedure – that is, all children are supposed to be diagnosed after the second or third test.

### Data analysis

Economic evaluation examines both the costs and the effects of two or more alternatives and thus provides information that can be used to optimize (usually: maximize) effectiveness in relation to the resources available [18]. Differences in costs (C) and effects (E) are related using incremental cost-effectiveness ratios (ICERs). Here, the ICER is defined as: (C_tracking_–C_no tracking_)/(E_tracking_–E_no tracking_). The ICER was used as the primary outcome measure in the economic analysis to compare tracking with no tracking. The results of the base case analysis and the sensitivity analyses are presented in the tables, a scatter plot, and a cost-effectiveness acceptability curve. As there is no evidence as to what is the maximum a decision-maker is willing to pay for an additional detected case of bilateral hearing impairment, a range of thresholds for cost-effectiveness was used, from €0 to €5,000.

## Results

### Decision-analytic model: base case analysis

The estimated effects and costs were combined in an ICER to calculate the incremental cost of detecting one additional case of bilateral hearing impairment. The base case analysis of the model is shown in Table 5. In the base case, the cost per case of bilateral hearing impairment detected was €40.12 for no tracking and €40.63 for tracking. In a hypothetical cohort of 100,000 newborns, the number of cases of bilateral hearing impairment detected with no tracking was 21, and with tracking 51. The ICER of tracking vs. no tracking was €1,697 per additional case of bilateral hearing impairment detected. Compared with no tracking, tracking resulted in more cases of bilateral hearing impairment detected as well as higher costs.

**Table 5:** Base case analysis of the model

**Intervention**	**Costs**	**Effects (cases detected)**	**ACER: Cost per case detected**	**ICER: Incremental cost per additional case detected**
Tracking	€40.63	0.00051	€79,667	-
No tracking	€40.12	0.00021	€191,048	€1,697

ACER: average cost-effectiveness ratio; ICER: incremental cost-effectiveness ratio.

### Decision-analytic model: sensitivity analyses

Table 6 shows the results of the univariate sensitivity analyses. It was found that the higher the probability of loss to follow-up before the second and consecutive tests with tracking, the higher the ICER; the lower the probability of loss to follow-up before the second and consecutive tests with no tracking, the higher the ICER; and the higher the costs of tracking, the higher the ICER. Overall, the results were relatively robust in the univariate sensitivity analyses: the ICER varied between €1,419 (probability of loss to follow-up before second test without tracking = 0.77) and €2,297 (probability of loss to follow-up before second test without tracking = 0.26) per additional case of bilateral hearing impairment detected.

**Table 6:** Univariate sensitivity analyses

							
*p_Ltfu2_t*^*a*^	*Costs (€) of no tracking*	*Costs (€) of tracking*	*Incremental costs (€)*	*Effects of no tracking*	*Effects of tracking*	*Incremental effects*	***ICER****(€/case detected)*
0.04	40.12	40.64	0.52	0.00021	0.00053	0.00032	1,628
0.07*	40.12*	40.63*	0.51*	0.00021*	0.00051*	0.00030*	1,697*
0.11	40.12	40.62	0.50	0.00021	0.00050	0.00029	1,775
*p_Ltfu2_nt*^*b*^	*Costs (€) of no tracking*	*Costs (€) of tracking*	*Incremental costs (€)*	*Effects of no tracking*	*Effects of tracking*	*Incremental effects*	***ICER****(€/case detected)*
0.26	40.19	40.63	0.44	0.00032	0.00051	0.00019	2,297
0.51*	40.12*	40.63*	0.51*	0.00021*	0.00051*	0.00030*	1,697*
0.77	40.04	40.63	0.59	0.00010	0.00051	0.00041	1,419
*p_Ltfu3_t*^*c*^	*Costs (€) of no tracking*	*Costs (€) of tracking*	*Incremental costs (€)*	*Effects of no tracking*	*Effects of tracking*	*Incremental effects*	***ICER****(€/case detected)*
0.04	40.12	40.64	0.52	0.00021	0.00053	0.00032	1,620
0.08*	40.12*	40.63*	0.51*	0.00021*	0.00051*	0.00030*	1,697*
0.12	40.12	40.63	0.51	0.00021	0.00050	0.00029	1,793
*p_Ltfu3_nt*^*d*^	*Costs (€) of no tracking*	*Costs (€) of tracking*	*Incremental costs (€)*	*Effects of no tracking*	*Effects of tracking*	*Incremental effects*	***ICER****(€/case detected)*
0.15	40.12	40.63	0.51	0.00024	0.00051	0.00027	1,853
0.29*	40.12*	40.63*	0.51*	0.00021*	0.00051*	0.00030*	1,697*
0.44	40.11	40.63	0.52	0.00018	0.00051	0.00033	1,551
*p_Ltfu4_t*^*e*^	*Costs (€) of no tracking*	*Costs (€) of tracking*	*Incremental costs (€)*	*Effects of no tracking*	*Effects of tracking*	*Incremental effects*	***ICER****(€/case detected)*
0.04	40.12	40.63	0.51	0.00021	0.00052	0.00031	1,663
0.07*	40.12*	40.63*	0.51*	0.00021*	0.00051*	0.00030*	1,697*
0.11	40.12	40.63	0.51	0.00021	0.00050	0.00029	1,740
*p_Ltfu4_nt*^*f*^	*Costs (€) of no tracking*	*Costs (€) of tracking*	*Incremental costs (€)*	*Effects of no tracking*	*Effects of tracking*	*Incremental effects*	***ICER****(€/case detected)*
0.09	40.12	40.63	0.51	0.00022	0.00051	0.00029	1,737
0.18*	40.12*	40.63*	0.51*	0.00021*	0.00051*	0.00030*	1,697*
0.27	40.12	40.63	0.51	0.00020	0.00051	0.00031	1,657
*c_tracking*^*g*^	*Costs (€) of no tracking*	*Costs (€) of tracking*	*Incremental costs (€)*	*Effects of no tracking*	*Effects of tracking*	*Incremental effects*	***ICER****(€/case detected)*
2.28	40.12	40.62	0.50	0.00021	0.00051	0.00030	1,654
4.55*	40.12*	40.63*	0.51*	0.00021*	0.00051*	0.00030*	1,697*
6.83	40.12	40.65	0.53	0.00021	0.00051	0.00030	1,740

**Figure 2:**
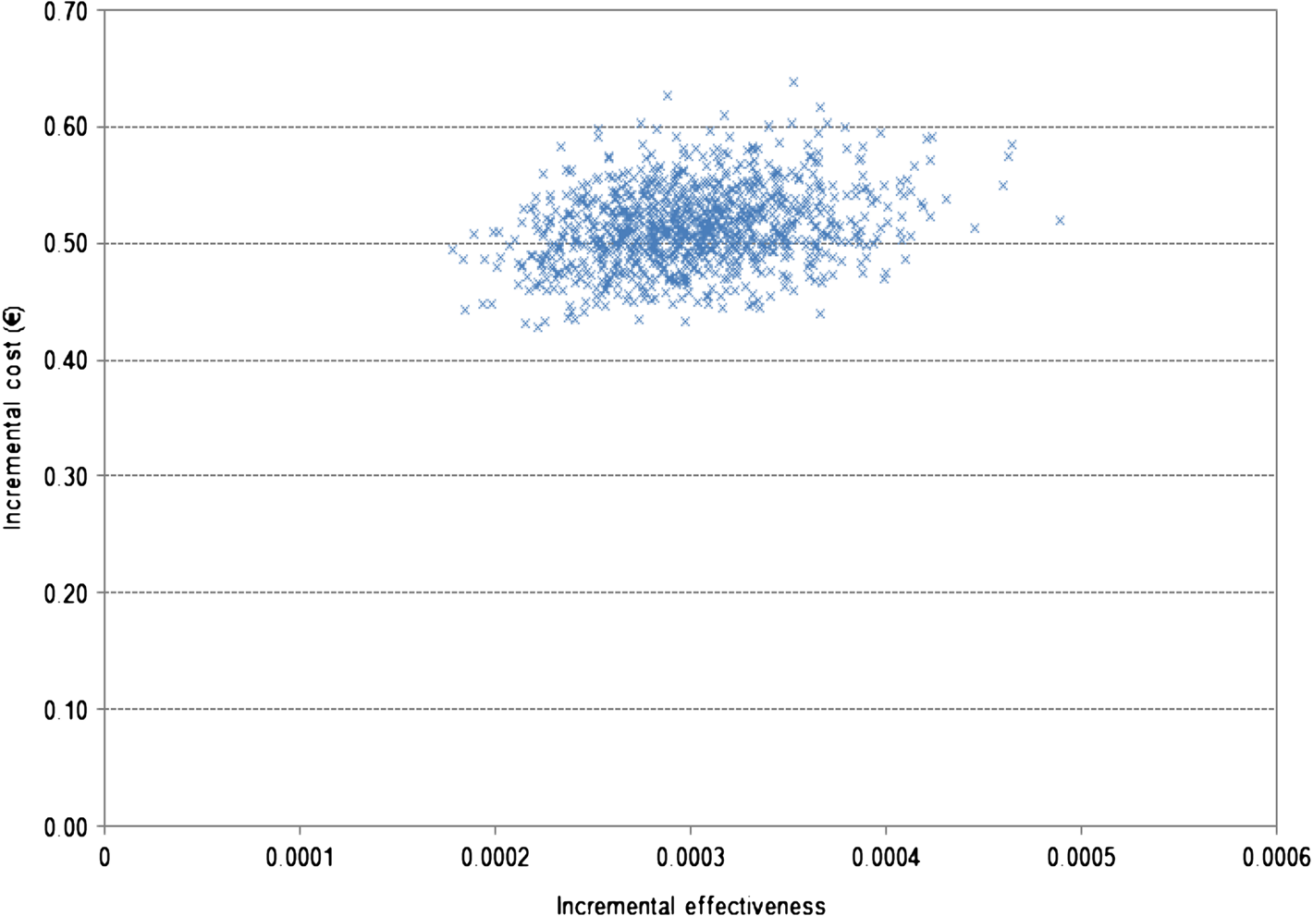
Incremental cost-effectiveness scatter plot for the model.

Figure 2 shows that the ICER in the second-order Monte Carlo simulation ranged from €1,060 to €2,769.

**Figure 3:**
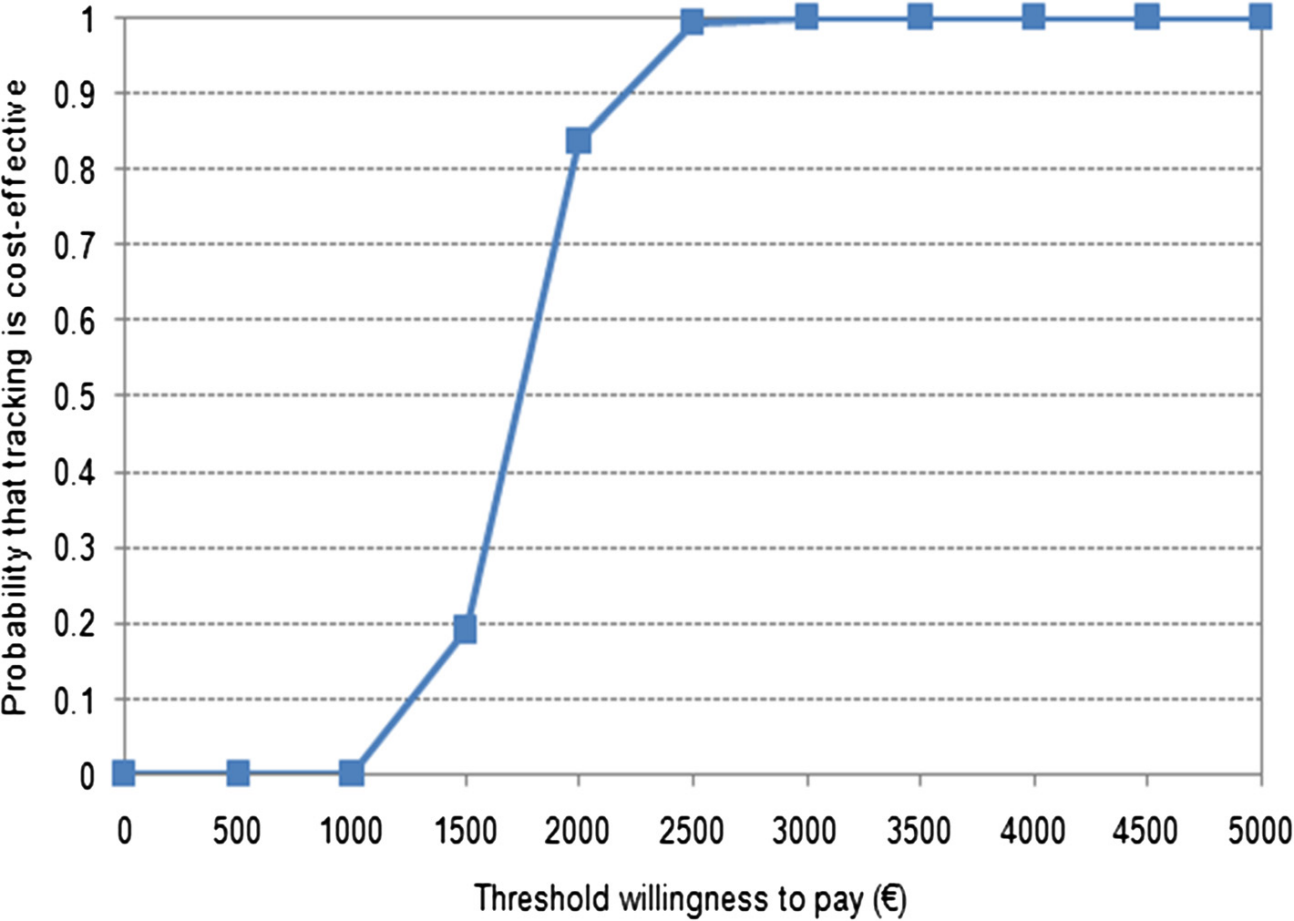
Cost-effectiveness acceptability curve for the model.

In Figure 3, the cost-effectiveness acceptability curve shows that, at a willingness to pay of €2,000 or €2,500 per additional case of bilateral hearing impairment detected, the probability that tracking is cost-effective was 83.8% or 99.5%, respectively.

The structural sensitivity analysis revealed that if all children were referred to pediatric audiologists and received a final diagnosis after the second or third test, the ICER would be €954 or €1,309 per case of bilateral hearing impairment detected.

## Discussion

Using a decision-analytic model based on data from Bavarian newborn hearing screening facilities, the cost-effectiveness of tracking newborns with bilateral hearing impairment in Bavaria was assessed. The costs of tracking in Bavaria – that is, €4.55 – compare well with those from a cost analysis of universal newborn hearing screening in Hesse, in which the costs of tracking were estimated at €4.00 [24]. According to a recent literature review [25], this is the first model to assess the cost-effectiveness of tracking within a newborn hearing screening programme; therefore the results of this model are not directly comparable with those of other models. However, with an ICER of €1,697 per additional detected case of bilateral hearing impairment, the implementation of a tracking system within a newborn hearing screening programme may be cost-effective, in particular with regard to the lifelong benefits of early detection and treatment, such as increased productivity owing to better language outcomes. In the pilot project, it is reported that from 2003 to 2008 there were 51 cases of confirmed bilateral hearing loss detected out of 73,332 infants screened, resulting in a rate of 0.70 per 1,000. That is higher than the rate of 0.51 per 1,000 with tracking used in the model. In the pilot project, 48% of the children with bilateral hearing impairment were followed up solely because of the existence of the tracking centre. Therefore, 27 cases of bilateral hearing impairment would have been diagnosed in the absence of the tracking programme, resulting in a rate of 0.5 per 1,000, compared with 0.31 per 1,000 in the decision-analytic model. However, the incremental number of children detected as a result of tracking is the same: 0.20 per 1,000.

Several economic evaluations have shown that the short-term cost-effectiveness of a newborn hearing screening programme depends not only on the diagnostic accuracy of the screening test procedure, but also on the ability to ensure follow-up of newborns with positive screening test results [20,26]. Tracking systems are, therefore, needed to ensure that early detection results in early intervention without unnecessary delays. Further studies are needed to evaluate the cost-effectiveness of tracking systems within newborn screening programmes.

The model used here has several limitations. First, it is assumed that, at the end of the four-stage test procedure, bilateral hearing impairment can be definitively confirmed or excluded. However, in contrast to other decision-analytic models which assume conditional independence, it could be considered that the probability that a newborn fails subsequent tests is conditional on having previous positive test results. However, this required the merging of data from two different newborn hearing screening programmes with different referral rates and rates of diagnosis conditional on referral. This merging of data could result in an underestimation of the number of cases detected relative to the experience of both newborn hearing screening programmes.

Second, some of the data used are taken from a Bavarian pilot project, and these data may therefore differ from data compiled subsequent to the nationwide implementation of newborn hearing screening in 2009, as well as data from other newborn hearing screening programmes in Germany. This may have implications for the generalizability of results; however, the issue of generalizability was addressed in the sensitivity analyses.

Third, only parameter and structural uncertainty was addressed via the sensitivity analyses, whereas methodological uncertainty (for example, discount rate, long time horizon) was not addressed, owing to a lack of long-term data. The cost-effectiveness of the intervention in different patient groups (uni- and/or bilateral hearing impairment) was not assessed because the target population was newborns with bilateral hearing impairment only, as is standard practice in Germany [4]. Thus, the analysis is rather conservative. If centralized tracking was extended to include unilateral referrals – some of which may indeed result in the diagnosis of bilateral hearing impairment – the incremental cost-effectiveness ratio would presumably be lower. A recent study found that children with unilateral hearing loss had worse language skills than their siblings with normal hearing [27]. However, more research is needed to clarify this issue.

Fourth, owing to a lack of adequate data, the time horizon was limited to the newborn hearing screening programme (initial hearing screening test and subsequent hearing tests or diagnosis) and a scenario analysis was not conducted.

Several studies have shown that the economic and disease burden of hearing impairment is high. The societal cost of severe to profound hearing loss over the lifetime of an affected person in the United States was estimated at US$297,000, mainly resulting from productivity losses (63%) and the requirement for special education (21%) [28]. Furthermore, permanent bilateral hearing impairment in children between 7 and 9 years of age was found to be associated with reduced health status and health-related quality of life compared with children with normal hearing [29], and an expected cost to society of about £14,000 in the preceding year of life [30]. Therefore, if a longer time horizon was taken into account, a transsectoral or even societal perspective on the effects on health-related quality of life would favour a newborn hearing screening programme which included tracking. Tracking may even be cost-saving from the perspective of public health services themselves (who pay for the tracking) once public expenditures for schooling etc. for children with special needs are taken into account. However, adequate and robust data on the long-term effects of tracking within newborn screening programmes with respect to costs and outcomes are lacking.

## Conclusions

Switching from no tracking to tracking costs €1,697 for each additional case of bilateral hearing impairment detected. If society is willing to pay at least €1,697 per additional case of bilateral hearing impairment detected, tracking can be recommended. Tracking may be even cost-saving in the long term if a high proportion of bilaterally hearing-impaired children go on to achieve normal language skills and so enjoy increased lifetime productivity, as a result of the early intervention thereby enabled. The cost-effectiveness of a newborn hearing screening programme does not depend only on the accuracy of the programme, but also on the ability to ensure follow-up of newborns that do not pass the initial hearing screening test and subsequent tests. Overall, then, this economic analysis is rather conservative because an outcome measure for the earliness of diagnosis was not included.

## Competing interests

The authors declare that they have no competing interests.

## Authors' contributions

AL carried out the design of the study and drafted the manuscript. All other authors participated in the design of the study. PM helped to conduct the decision-analytic modelling and to draft the manuscript. IB and UNR provided the data that were not openly available. All authors read and approved the final manuscript.

## Pre-publication history

The pre-publication history for this paper can be accessed here:

http://www.biomedcentral.com/1472-6963/12/418/prepub
